# External validation based on transfer learning for diagnosing atelectasis using portable chest X-rays

**DOI:** 10.3389/fmed.2022.920040

**Published:** 2022-07-22

**Authors:** Xiaxuan Huang, Baige Li, Tao Huang, Shiqi Yuan, Wentao Wu, Haiyan Yin, Jun Lyu

**Affiliations:** ^1^Department of Neurology, The First Affiliated Hospital of Jinan University, Guangzhou, China; ^2^Department of Clinical Research, The First Affiliated Hospital of Jinan University, Guangzhou, China; ^3^Department of Radiology, The First Affiliated Hospital of Jinan University, Guangzhou, China; ^4^School of Public Health, Xi’an Jiaotong University Health Science Center, Xi’an, China; ^5^Department of Intensive Care Unit, The First Affiliated Hospital of Jinan University, Guangzhou, China; ^6^Guangdong Provincial Key Laboratory of Traditional Chinese Medicine Informatization, Guangzhou, China

**Keywords:** atelectasis, transfer learning, ResNet, artificial intelligence (AI), ICUs

## Abstract

**Background:**

Although there has been a large amount of research focusing on medical image classification, few studies have focused specifically on the portable chest X-ray. To determine the feasibility of transfer learning method for detecting atelectasis with portable chest X-ray and its application to external validation, based on the analysis of a large dataset.

**Methods:**

From the intensive care chest X-ray medical information market (MIMIC-CXR) database, 14 categories were obtained using natural language processing tags, among which 45,808 frontal chest radiographs were labeled as “atelectasis,” and 75,455 chest radiographs labeled “no finding.” A total of 60,000 images were extracted, including positive images labeled “atelectasis” and positive X-ray images labeled “no finding.” The data were categorized into “normal” and “atelectasis,” which were evenly distributed and randomly divided into three cohorts (training, validation, and testing) at a ratio of about 8:1:1. This retrospective study extracted 300 X-ray images labeled “atelectasis” and “normal” from patients in ICUs of The First Affiliated Hospital of Jinan University, which was labeled as an external dataset for verification in this experiment. Data set performance was evaluated using the area under the receiver operating characteristic curve (AUC), sensitivity, specificity, and positive predictive values derived from transfer learning training.

**Results:**

It took 105 min and 6 s to train the internal training set. The AUC, sensitivity, specificity, and accuracy were 88.57, 75.10, 88.30, and 81.70%. Compared with the external validation set, the obtained AUC, sensitivity, specificity, and accuracy were 98.39, 70.70, 100, and 86.90%.

**Conclusion:**

This study found that when detecting atelectasis, the model obtained by transfer training with sufficiently large data sets has excellent external verification and acculturate localization of lesions.

## Introduction

Atelectasis, as the most common postoperative pulmonary complication (PPC) (1), is also the most common disease in intensive care units (ICUs), and is often accompanied by pneumothorax, pleural effusion, pulmonary edema, and other pulmonary diseases, and requires reintubation within 48 h after a complication. Portable chest radiography (2) is one of the most common non-invasive radiological tests for rapid and straightforward atelectasis detection in ICUs. The main direct signs (3) of atelectasis on chest radiographs include defect migration, parenchymal opacity with unbroken linear boundaries, and vascular displacement. Indirect signs (4) have ipsilateral diaphragmatic elevation, hilar removal, heart involvement, and mediastinum and trachea dysfunction; however, it is difficult to rapidly distinguish the characteristics of early chest radiographs of patients with atelectasis from pleural effusion and lung consolidation with increased density. With the rapid development of deep-learning technology, the convolutional neural network (5) extracts inherent characteristics from medical image data for classification and recognition based on images much more effectively than do traditional recognition algorithms. The problem of inaccurate diagnoses caused by the continuous increase in the number of chest X-rays that exceeds the increase in the number of radiologists has somewhat been solved. Because previous studies have developed chest X-ray diagnostic algorithms based on deep neural networks, 14 basic lung diseases can be diagnosed.

The present study was the first to extract large-scale positive atelectasis data from the MIMIC-CXR-JPG database (6–8), an intensive care medical information database, to obtain a model with high accuracy and obtain reliable external validation. The purpose of this study was to determine the feasibility of transfer learning methods for detecting atelectasis using portable chest X-rays based on large data sets. In addition, this study used multi-center data set for the first time to realize the early diagnosis and prediction of bedside portable chest radiographs and obtained good external validation.

## Materials and methods

### Data source

#### Training and testing cohorts

In this study, 14 categories that were clearly diagnosed as “positive” or “negative” were obtained from the MIMIC-CXR-JPG database using the open-source tagging tools NegBio9 (9) and CheXpert10 (10) (473,057 chest radiographs and 206,563 text reports). There were 45,808 chest radiographs labeled “atelectasis” and 75,455 chest radiographs labeled “no finding.” A total of 60,000 images were extracted, including positive images labeled “atelectasis” and positive X-ray images labeled “no finding,” and blank text was excluded. The data were defined as “normal” and “atelectasis” with an even distribution. At the same time, the data set was classified and randomly divided into three sets (training, validation, and testing) at a ratio of about 8:1:1. The MIMIC-CXR-JPG common database was used as the internal testing data of this experiment.

#### External validation cohort

**FIGURE 1:**
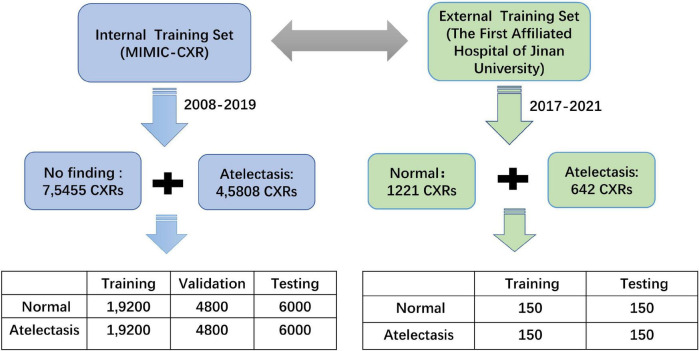
Data flow design of the internal and external datasets.

An external testing data set was developed in this study, with primary image data from ICUs patients at The First Affiliated Hospital of Jinan University, from which data during 2017–2021 were randomly selected by three senior attending physicians for a definitive diagnosis of the patients with atelectasis, who found no apparent abnormalities in the chest radiological image data, and professional radiologists carried out a review of the random tag data set. After excluding the data of poor posture placement and unclear diagnoses, 300 images with complete labels were finally extracted: 150 with atelectasis and 150 without abnormalities. The flow-chart of the training data creation is shown in Figure 1.

### Experimental environment

All experiments were performed using the Ubuntu 20.04 64-bit operating system. For the training process of the CNN model, MATLAB language was adopted as the programming environment. The specific software and hardware configuration are listed in Table 1.

**TABLE 1:** Hardware and software configuration.

Experimental environment	Configuration instructions	
Hardware environment	CPU	Intel (R) Xeon 5218 16C 2.3 GHz
	GPU	NVIDIA TESLA V100, 32 GB
	Memory	32 GB
Software environment	Operating system	Ubuntu 20.04
	Programming environment	MATLAB 2021a

### Data preprocessing

This study processed both internal and external testing cohorts. The frontal chest images from the MIMIC-CXR-JPG database had a resolution of 256 × 256 × 1 pixels, while those from The First Affiliated Hospital of Jinan University were 512 × 512 × 3 pixels. Since the image resolutions of the two data sets had different heights and widths, the image data from the two other sources had to be standardized. We achieved this by automatically cropping the chest region and adjusting it to 224 × 224 × 3 pixels in order to fit the model input resolution more conveniently. At the same time, the image was randomly scaled horizontally and vertically, and processing methods were cut and shifted to enhance the data processing (11) in order to further optimize the verification and evaluation of the testing results.

### Model

**FIGURE 2:**
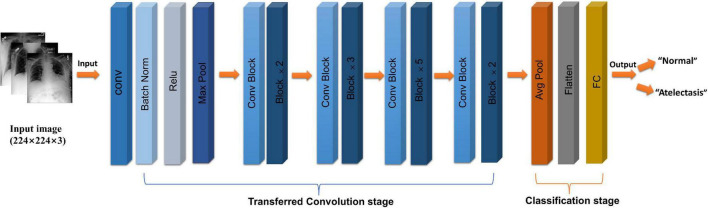
Flow chart of ResNet50 (CoNV is convolution operation, Batch Norm is Batch regularization processing, ReLU is activation function, MAXP00L and AvgPOOL are two pooling operations, including convolution transformation stage, and classification stage, respectively).

The selected study model was the ResNet50 (12) network model, which is based on the residual error learning method from the VGG19 improved classification model. Based on the existing training network depth, a more-optimized residual learning framework was put forward, not only to solve the problem of the gradient disappearance and explosion (13), but also to further deepen the network depth. The problem of network performance degradation is also avoided. ResNet50 covers 49 convolutional layers and one full connection layer, retains the convolutional layer with a core size of 7 × 7 in VGG19 (14) to learn more features, and uses the maximum pooling layer for downsampling. In addition, there are five stages and two significant boards in the ResNet50 network. Stage 0 has a simple structure and is mainly used for the preprocessing of input images. It has gone through the convolutional layer, batch normalization, and ReLU activation function (12). The next four stages are composed of a bottleneck and have similar structures. After continuous convolution operation of residual blocks, the number of channels in the image pixel matrix becomes deeper and deeper, then passes through the flatten layer, and finally input into the full connection layer and output the corresponding category probability through SoftMax layer, the typical features of the image are automatically extracted, and the last constitute a classifier to divide the image into “normal” and “atelectasis.” The specific network architecture is shown in Figure 2.

### Training strategy

Considering the extensive data set analyzed in this study, a general machine-learning method called transfer learning was used to improve the rate and performance of model learning. Transfer learning (15, 16) can transfer the knowledge learned by the model from the source domain to another target domain, so that the model can better acquire the understanding of the target domain, and improve the speed and simplicity of the learning, compared with the initial training network that uses randomly initialized weights. This study used a pretrained ResNet50 network model to randomly divide the data into the training (80%) and validation (20%) sets, and the same pretreatment operation was adopted. Since the chest radiography images were asymmetric (16), we adopted random undersampling technology and adaptive moment estimation (Adam) using vector momentum, which was adapted to increase the convergence speed. Our model was trained with a 0.0001 learning rate, minimum batch size of 64, and maximum of 8 epochs, in order to achieve the maximum number of 7,200 iterations. The training lasted 105 min and 6 s. By fine-tuning the experimental parameters, the best experimental results were obtained. The training effect is shown in Figure 2.

### Performance analysis

In the experiments, the area under the receiver operating characteristic curve (AUC) (17) and the accuracy, specificity, and sensitivity (Eqs. 1, 2, and 3 below) were used as evaluation indicators (18). A larger AUC indicates that the prediction result is closer to the actual situation, and hence better model performance. Through the following formula, we can get the following indicators, respectively: false positives (FP), true negatives (TN), true positives (TP), and false negatives (FN). The confusion matrices were calculated from the following indexes, which can further help to analyze the model performance and calculate the above evaluation values.


(1) Accuracy=(TP+TN)/(TP+FN+TN+FP)

(2) Specificity=TN/(TN+FP)

(3) Sensitivity=TP/(TP+FN)

### Visualization

Based on the model obtained by the experimental training, Grad-CAMs (19, 20)can be used to generate a gradient class activation map. Grad-CAMs generated the class activation map and highlighted the areas that are important for the classification of the image, which not only provides insight into the nature of the black box of the model (16), but is also helpful for obtaining the key feature extraction of images and enables predictions of the classification decision interpretation model.

## Results

**FIGURE 3:**
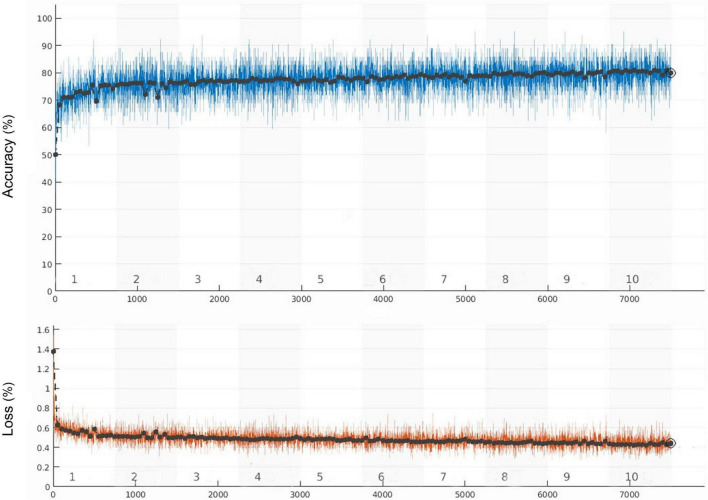
Training accuracy and training loss curves (the black curve and blue curve represent the accuracy of training set and verification set, respectively, the orange curve represents the loss).

The whole training process took 105 min and 6 s. The learning curve of the model is shown in Figure 3, and the verification accuracy reached 79.91%. The partial accuracy curve reached 80% when the training was completed, and the loss rate decreased significantly to below 40%.

**FIGURE 4:**
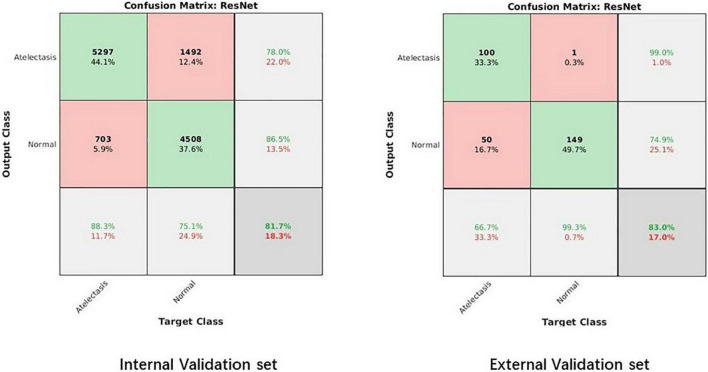
The confusion matrix of the internal and external validation sets.

**FIGURE 5:**
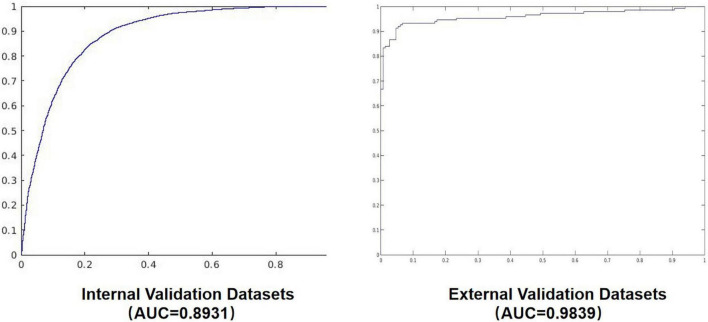
The AUC diagram of the internal and external validation sets.

The confusion matrix results of the model on the testing and validation cohorts were calculated and shown in Figure 4. The testing cohort was distributed in a 2 × 2 matrix according to the labeled labels and the predicted results. Each square represents the ratio of predicted positives to actual positives. Total data volume and prediction are shown for each level. The trained model can classify the testing cohort at an accuracy of 81.70%, and the specificity and sensitivity were 88.30 and 75.10%, respectively. An accuracy of 86.90% was obtained by classifying the external validation set, and the corresponding specificity and sensitivity in the calculations were 100 and 70.70%, respectively. Table 2 lists all the evaluation indexes obtained in the internal and external testing cohort of this experiment. By calculating the above scores, the confusion matrixes of the internal and external testing cohorts were obtained, which could help to get TPs, TNs, FPs, and FNs. Meanwhile, the AUC was used as the evaluation index, with the vertical axis representing the true category rate. The horizontal and vertical axes represented the FP and TP rates, respectively, and the ROC curve was drawn. Larger AUC values indicate that the model prediction result is closer to the actual situation. The AUCs of the internal and external testing cohorts obtained in the model training were 88.57 and 98.39%, respectively, as shown in Figure 5.

**TABLE 2:** Accuracy, sensitivity, specificity, AUC scores of internal and external datasets.

Datasets	Accuracy	Sensitivity	Specificity	AUC scores
Internal validation datasets	81.70%	75.10%	88.30%	89.31%
External validation datasets	85.30%	70.70%	100.00%	98.39%

**FIGURE 6:**
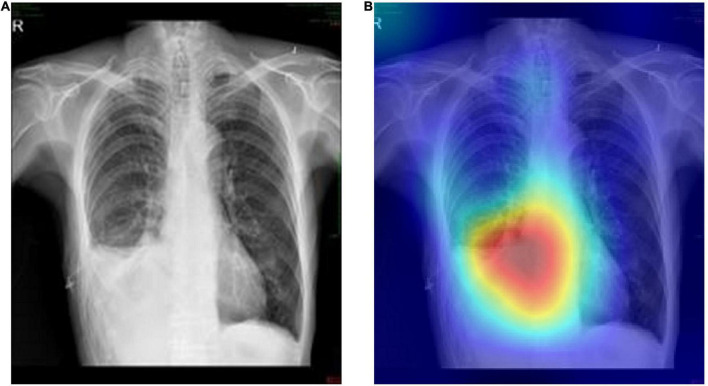
Representative cases in the external testing sets. **(A)** Example of a true-positive case, that is the original chest radiograph of atelectasis with pleural effusion in ICUs. **(B)** Grad-CAM heatmap of class activation derived from model prediction classification.

On this basis, the Grad-CAMs map was drawn to predict the deep model. Figure 6 shows that the proposed model can accurately distinguish between normal chest radiographs and those with atelectasis. The red area on the chest radiographic heat map offers the critical area for the machine to determine its classification (21). Randomly generated heat maps focused on the lungs and heart.

## Discussion

Atelectasis remains a significant challenge for physicians in general anesthesia and ICUs treatment and diagnosis. Undiagnosed or late-diagnosed atelectasis can have a significant mortality risk (22). From a pathological point of view (3), atelectasis mainly manifests as reversible alveolar or lobe collapse, which is generally caused by obstruction of the affected alveoli in the airways, resulting in damage to the exchange of carbon dioxide and oxygen. According to preliminary studies, almost all patients undergoing major surgery will present with some degree of atelectasis (23, 24). Typically 2–4% of elective thoracic surgeries and 20% of emergency surgeries are related to PPCs, among which atelectasis is the most common respiratory complication. Without timely early diagnosis and intervention, a series of serious and often fatal complications will occur as the disease progresses. Eventually, due to decreased lung compliance, hypoxemia, decreased pulmonary vascular resistance, hypoxemia, postoperative infection, diffuse alveolar injury, respiratory failure, or even death (in extreme cases) may occur. So far, X-ray imaging has always been an essential means of atelectasis diagnosis, and portable chest X-ray in ICUs (25) is a rapid and straightforward method for the early diagnosis of atelectasis. This is especially true among ICUs patients with respiratory and hemodynamic parameters within a normal range, and where the direct signs of atelectasis mostly appear on chest radiograph crack deviations (3, 4, 26), parenchymal turbidities, linear boundary, and vascular displacements, among which increased density of dysfunctional lung areas is the most-obvious manifestation of atelectasis. In order to help ICUs doctors diagnose atelectasis early using portable chest X-ray, we established a model of bedside chest X-rays for detecting atelectasis by applying transfer learning based on the ResNet50 convolutional network, and used the explicit atelectasis data extracted from the MIMIC-CXR-JPG database as an internal testing cohort, which yielded an accuracy of 81.70%. This result was further externally verified using ICUs atelectasis image data rescreened and relabeled by doctors at The First Affiliated Hospital of Jinan University, achieving an accuracy rate of 86.90%. Obviously, the process of data relabeling is one of the main reasons for the increased accuracy of external validation in this study compared with previous studies.

Data enhancement and transfer learning were simultaneously adopted in this study to improve the accuracy of image classification and avoid overfitting. The ResNet50 network model parameters were obtained through the migration study less, high precision, deep residual layer network structure is complex, which solves the problem of low efficiency based on large training data sets and makes training more precise. Calculating the specificity, sensitivity, accuracy, and AUC revealed that the training model was highly robust (27), which provided external verification, with values of 100.00, 70.70, 86.90, and 98.39%, respectively. Finally, the features extracted from the training images were visualized by a heat map displaying the lung and the region near the heart. In addition, the novelty of this study was highlighted by the application of the transfer learning method to chest X-ray atelectasis examinations, and its reliable external validation.

This study had some limitations. Firstly, we used all the atelectasis image data sets during 2011–2017 in the MIMIC-CXR-JPG database, which is large but only provided relatively limited patient information, such as gender, age, and diagnostic test, and the clinical backgrounds of patients were unavailable. Atelectasis diagnoses could therefore only be labeled according to the diagnostic test, and whether it was associated with other pulmonary complications remains to be determined. We will further attempt to establish a more practical model combined with the experience of clinical practice, use more diverse neural network learning algorithms and network models, and make horizontal comparison with other more advanced networks, to classify atelectasis in more detail, with a view to providing greater assistance in early clinical intervention, diagnosis and treatment. Secondly, the case-control design used in this study artificially increased the prevalence of atelectasis by using positive data collected from the MIMIC-CXR-JPG database and The First Affiliated Hospital of Jinan University, thus overestimating the positive predictive value compared with the clinical reality (28). In addition, the data sets of internal and external validation included in this paper are random and uniform data, which satisfies the ideal comparison of data to a certain extent. Queue design will therefore be used in the future to obtain more-reliable actual tags. Thirdly, our internal and external testing cohorts were basically derived from portable chest X-ray images from ICUs and the results might not be applicable to outpatients and general patients. Moreover, the image data in the MIMIC-CXR-JPG database (6, 29) were derived from foreign databases, and there were some differences in diagnostic reporting and standards. There was some heterogeneity in the atelectasis data obtained from The First Affiliated Hospital of Jinan University based on the external validation, and so the results obtained should be considered exploratory only.

In the future, we will attempt to explore more cutting-edge and optimized AI models for portable chest X-ray diagnoses of acute and severe pulmonary complications such as atelectasis, and further promote precision medicine (30, 31), to allow the application of machine learning in clinical imaging diagnosis to realize human-machine mutual assistance and true generalization.

## Conclusion

In summary, this study found that when detecting atelectasis, a model obtained by training with sufficiently large data sets exhibited better external verification and can better help ICUs doctors to diagnose atelectasis and implement interventions early.

## Data availability statement

The raw data supporting the conclusions of this article will be made available by the authors, without undue reservation.

## Ethics statement

The studies involving human participants were reviewed and approved by the IRB of the First Affiliated Hospital of Jinan University. The patients/participants provided their written informed consent to participate in this study. Written informed consent was obtained from the individual(s), and minor(s)’ legal guardian/next of kin, for the publication of any potentially identifiable images or data included in this article.

## Author contributions

XH created the study protocol, completed the main experimental training, and wrote the first manuscript draft. BL provided clinical guidance and critically revised the manuscript. TH assisted with the study design and performed data collection. SY and WW participated in the analysis and interpretation of data. HY assisted with manuscript revision and data confirmation. JL contributed to data interpretation and manuscript revision. All authors contributed to the article and approved the submitted version.

## Conflict of interest

The authors declare that the research was conducted in the absence of any commercial or financial relationships that could be construed as a potential conflict of interest.

## Publisher’s note

All claims expressed in this article are solely those of the authors and do not necessarily represent those of their affiliated organizations, or those of the publisher, the editors and the reviewers. Any product that may be evaluated in this article, or claim that may be made by its manufacturer, is not guaranteed or endorsed by the publisher.

## Abbreviations

MIMIC-CXR: medical information mart for intensive care chest X-ray
AUC: area under the curve
ROC: curves receiver operating characteristic curves
ICUs: intensive care units
AI: artificial intelligence.
